# Prevalence of bone fractures among children and adolescents with attention-deficit/hyperactivity disorder: a systematic review and meta-analysis

**DOI:** 10.1186/s12887-021-02821-x

**Published:** 2021-08-19

**Authors:** Hoda Seens, Shirin Modarresi, Joy C MacDermid, David M Walton, Ruby Grewal

**Affiliations:** 1 Health and Rehabilitation Sciences, Western University, London, ON Canada; 2 Windsor University School of Medicine, Cayon, Saint Kitts and Nevis; 3 School of Physical Therapy, Western University, London, ON Canada; 4 Roth McFarlane Hand and Upper Limb Centre, St. Joseph’s Health Care London, London, ON Canada; 5 Schulich School of Medicine and Dentistry, Western University, London, ON Canada

**Keywords:** Attention-deficit/hyperactivity disorder, Bone fracture, Children, Adolescents, Injury, Accident

## Abstract

**Background:**

Attention-deficit/hyperactivity disorder (ADHD) is a significant neurodevelopment disorder among children and adolescents, with 5 % prevalence. Bone fractures account for 25 % of accidents and injuries among all children and adolescents. Considering the characteristics of inattention, hyperactivity, and impulsivity in children with ADHD, it is critical to examine bone fractures among these children. The objective of our meta-analysis was to determine the prevalence of bone fractures among children and adolescents with ADHD.

**Methods:**

We completed a systematic review and meta-analysis using an electronic search of the following databases: CINAHL, EMBASE, PsycINFO, PubMed, and Scopus. The search terms used were: “attention deficit hyperactivity disorder OR attention deficit disorder” and “bone fracture*.” We included studies examining patients 18 years or younger who were diagnosed with ADHD and tracked (prospectively or retrospectively) for five or more years. Effect size (es), using a random effects model, was calculated. We registered the review protocol with PROSPERO (CRD42019119527).

**Results:**

From 445 records retrieved, 31 full text articles were reviewed and 5 articles met inclusion criteria for meta-analysis. The summary es revealed the prevalence of bone fractures among children and adolescents with ADHD to be 4.83 % (95 % CI: 3.07–6.58 %). The location of bone fractures, using a subset of data, showed a distribution of 69.62 %, 22.85 %, and 7.53 % in the upper limbs, lower limbs, and other anatomical regions, respectively. Another subset of studies revealed a 2.55-fold increase in the prevalence of fractures among the children with ADHD compared to their counterparts.

**Conclusions:**

Awareness of these findings is critical to physicians, parents, and policy makers to create safe environments and provide supports in order to optimize the health and safety of children and adolescents with ADHD.

## Background

Attention-deficit/hyperactivity disorder (ADHD) can be described as a neurodevelopment disorder of impairing inattention, motor hyperactivity, and impulsivity with an onset in childhood and difficulties continuing into adulthood [1]. There has been great variability in the reported prevalence of ADHD between studies, explained mainly by differences in their methodology [2]. One such methodological variation is differences in the definition of ADHD used, such as those between the Diagnostic and Statistical Manual of Mental Disorders (DSM) versus the International Classification of Diseases (ICD) classifications. Other reasons for the discrepancy originate from the demographic characteristics of the population under study, such as age and gender, as well as the source of information [3]. Overall, according to DSM-5 [4], the prevalence of ADHD is estimated to be 5 % and the male to female ratio is nearly 2:1 among children.

Bone fractures account for nearly 25 % of all accidents and injuries among children, with fractures of the distal radius being the most common [5]. Overall, extremity fractures are one of the most common reasons of children’s hospital admission [6]. The three main causes of children’s extremity fractures are accidental trauma, non-accidental trauma (that is, child abuse), and pathological bone conditions [7]. Among the accidental reasons of these fractures are falling from a height or falling while running, motor vehicle accidents (such as being hit by a car as a pedestrian), fighting, and recreational activities (such as riding a bicycle) [7].

One may assume that symptoms of ADHD, such as hyperactivity, ought to predispose children and adolescents diagnosed with ADHD to a greater number of accidental injuries [8]. This notion is supported by evidence from Brehaut, Miller, Raina, and McGrail [9] who report a 1.5-fold increase in the risk of injury in children with behavioral disorders.

Considering the increased risk of injuries among children with ADHD and the high prevalence of bone fractures among children, it is essential to examine bone fractures in children and adolescents with ADHD. To the authors’ knowledge, no review of these findings has been conducted. The purpose of this review is to systematically assess the available studies and to synthesize their findings as a meta-analysis.

## Methods

The authors have adhered to the Preferred Reporting Items for Systematic Reviews and Meta-Analyses (PRISMA) statement guidelines in completing this systematic review and meta-analysis [10]. The review has also been registered with PROSPERO (CRD42019119527).

### Search strategy

The Cochrane Library and PROSPERO were searched to ensure that the systematic review of fractures among children and adolescents with ADHD had not previously been completed. Then, an electronic search of the following databases was conducted to retrieve studies: CINAHL, EMBASE, PsycINFO, PubMed, and Scopus. The following search terms were used: “attention deficit hyperactivity disorder OR attention deficit disorder” and “bone fracture*.” There were no restrictions set on language or publication dates of retrieved articles. To supplement the electronic search, reference lists from key articles and other systematic reviews on the topic of ADHD and injuries were identified and assessed for articles meeting the systematic review criteria.

### Eligibility criteria

In order to fulfill inclusion requirements, studies had to meet five general criteria. First, the age range for the population under study was set at 18 years old and younger. If a study’s population age range had 60 % or greater overlap with this age range, it would be included. For example, a study whose population was ages six to 17 years would qualify but a study whose population was 40 years or younger would be excluded.

The second criterion for inclusion was a diagnosis of ADHD, which must have been made using recognized diagnostic criteria, such as the DSM or ICD classifications, or the use of prescription medication for the treatment of ADHD in the population. For example, a study that identified participants by relying on a survey to classify children as hyperactive without standard diagnosis of ADHD would be excluded. On the other hand, a study that used the prescription of methylphenidate to indicate the presence of ADHD would be included. This latter decision was made because medications used to treat ADHD are not typically used off-label in children or adolescents for other purposes; therefore, an underlying diagnosis of ADHD is likely present if children or adolescents are being prescribed methylphenidate [11].

The third inclusion criterion concerned the type of study and study methodology. To be included in the meta-analysis, the study must have provided a sample of patients with ADHD followed (prospectively or retrospectively) over a period of five or more years with a subset that have experienced bone fractures. The patients with ADHD could not be part of a specific population, such as Medicaid patients or have fractures sustained exclusively as a result of physical abuse. However, the population was not restricted to any geographic location or setting (for example, inpatient or outpatient records were acceptable).

The fourth inclusion criterion was the article type. To be considered in the review, articles had to be published in peer-reviewed journals as full-text manuscripts. Conference abstracts and commentaries were excluded from the review. Finally, the fifth inclusion criterion was that the study population was a unique population that had not been covered by any other article in the review. Studies that examined the same dataset or group of patients through various articles were only included once in the meta-analysis. Additionally, it should be noted, that this review was on bone fractures in human subjects only; therefore, studies conducted exclusively on animal models or human dental fractures were excluded.

### Study selection

The resulting articles from the electronic search were entered into Mendeley (version 1.19.2) where duplicates were removed. Then articles were assessed based on their abstracts to exclude entries that did not meet the inclusion criteria. Subsequently, full-text articles were retrieved for the remaining entries and examined by two independent reviewers (H.S. and S.M.) to evaluate inclusion into the systematic review and meta-analysis.

### Data extraction

A data extraction form was created to extract key elements of the study, including findings that could be used to calculate fracture prevalence among subjects with ADHD. Data extraction included the following: (1) author, (2) year, (3) country, (4) study setting, (5) study design, (6) duration, (7) sample size of ADHD group, (8) sample size of control group, if applicable, (9) age range, (10) ADHD diagnosis method, (11) fracture identification method, and (12) fracture locations, if specified.

### Quality assessment

Two independent reviewers (H.S. and S.M) conducted quality assessment of the studies that were included in the meta-analysis using the Joanna Briggs Institute (JBI) critical appraisal tool for prevalence studies [12]. The checklist includes nine questions that are marked as “yes,” “no,” “unclear,” or “not applicable.” The authors of this meta-analysis agreed to translate the checklist into a point system by assigning one point for any question marked “yes,” no points to questions marked “no” or “unclear,” and not to count any questions marked as “not applicable” in the final calculation.

### Data analysis

Meta-analysis was completed using a Microsoft Excel tool developed by Neyeloff, Fuchs, and Moreira [13]. The sample size of each study was the number of patients diagnosed with ADHD. The number of events was the number of fractures experienced within each study’s ADHD group. The prevalence of fractures among this population was the calculated effect size. Neyeloff, Fuchs, and Moreira [13] warn that combining prevalence rates cannot be called “effects” because there is not an intervention causing an effect. Rather, they state that this rate should be named “single group summary” and refer to it simply as “outcome,” which they abbreviate as “es, effect size.” For the purpose of simplicity, this meta-analysis will refer to the outcome (prevalence of fractures) as es.

The authors decided to use a random effects model to calculate the es of the meta-analysis for several reasons. A random effects model allows for the assumption that factors other than error or chance exist within and between studies. It also allows for the assumption that the studies used are a random sample of a hypothetical population of studies. Additionally, a random effects approach allows for generalizations to be made beyond the studies included in the meta-analysis [14]. JBI encourages the use of a random effects model that is presented with a 95 % confidence interval (CI) for prevalence meta-analyses [14].

The level of heterogeneity or “variation in true effect sizes underlying the different studies” [15, p. 1158] included in the meta-analysis was assessed using the I^2^ measure. Higgins, Thompson, Deeks, and Altman [16] recommend the use of I^2^ as a measure of consistency between studies, especially when a meta-analysis consists of a small number of studies. I^2^ is total variation (in percentage) across studies due to heterogeneity and not due to chance [14]. Heterogeneity is expected in meta-analysis [15] especially when it comes to studies in medicine [17].

## Results

### Search results

**Fig. 1:**
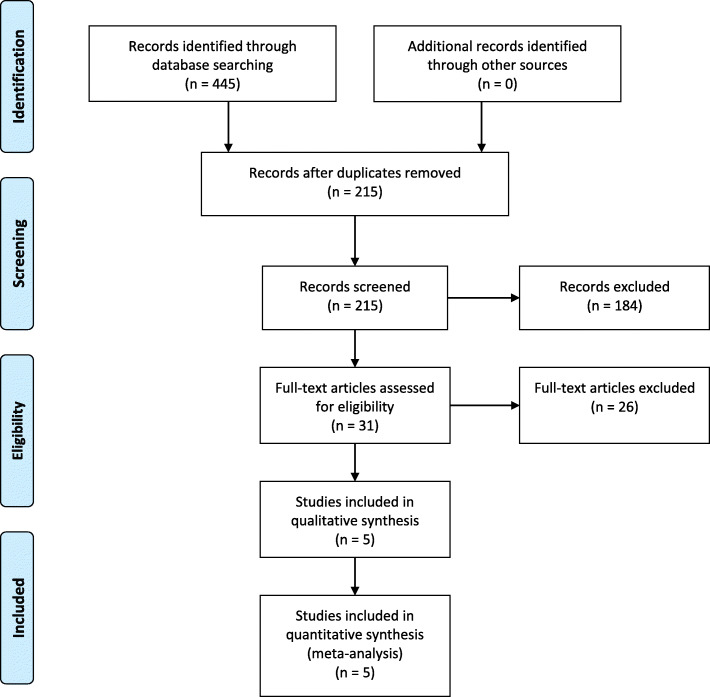
PRISMA flow diagram for study inclusion

The initial database search resulted in 445 articles (Fig. 1). Of these articles, 230 were found to be duplicates and removed. The remaining 215 articles were reviewed for inclusion based on their abstracts, from which 184 articles were excluded. Then, the remaining 31 records were retrieved for analysis of the full text, of which five met the inclusion criteria and were included in the systematic review and meta-analysis.

### Excluded studies

As mentioned above, 184 articles were excluded after an examination of their abstracts. Common reasons for exclusion at this point were articles that exclusively examined dental fractures in the ADHD population, the effects of brain injury on the development of ADHD, non-human models, the effects of ADHD medication on fractures, and fractures as a result of abuse. It was also possible to exclude, based on abstracts, several studies that did not meet the age inclusion requirements.

The next stage of study exclusion was based on a full-text appraisal of the remaining 31 articles (completed by H.S. and S.M). Of these, 26 articles were excluded based on not meeting the inclusion criteria of age (*n* = 4), ADHD diagnosis (*n* = 8), study type (*n* = 10), article type (*n* = 3), and being a population examined by another study that was already included (*n* = 3). Two of these articles were excluded for two reasons each (Table 1).

**Table. 1:** Summary of excluded articles, with reasons

Article	Exclusion Criteria					Explanation
	**Age**	**ADHD Diagnosis**	**Article Type**	**Study Type**	**Repeat Population**	
Ayaz, 2015 [18]				x		Point-in-time data
Barkley, 2001 [8]			x			Commentary
Cairney, 2014 [19]			x			Commentary
Chen, 2017 [20]					x	Same population as Chou, 2014
Duramaz, 2018 [21]		x				Tool used assessed impulsive behavior (not ADHD diagnosis)
Erdogan, 2014 [22]				x		All patients have fractures (case-control)
Guo, 2016 [23]					x	Same population as Chou, 2014
Guy, 2016 [24]				x		Assessed Medicaid patients only
Hellgren, 1993 [25]		x				Mixed patient population (cannot isolate ADHD)
Hurtig, 2016 [26]		x				Assessed hyperactivity and symptoms consistent with ADHD (not ADHD diagnosis)
Lu, 2017 [27]		x				ADHD diagnosed only as a comorbid condition with Tourette Syndrome
Merrill, 2009 [28]	x					Population 0–65 years
Ozer, 2010 [29]				x		All patients have fractures (case-control)
Pastor, 2006 [30]				x		Point-in-time data
Perry, 2016 [31]	x					Population 0–40 years
Prasad, 2018 [32]				x		Point-in-time data
Rowe, 2004 [33]				x		Point-in-time data
Schermann, 2019 [34]	x					Population of military recruits
Schermann, 2018 [35]	x					Population of military recruits
Siwani, 2014 [36]				x		All patients have fractures (case-control)
Tai, 2013 [37]		x			x	All hyperkinetic disorders are included; Same population as Chou, 2014
Uslu, 2008 [38]		x				Tool used assessed impulsive behavior (not ADHD diagnosis)
Uslu, 2007 [39]		x				Tool used assessed impulsive behavior (not ADHD diagnosis)
van den Ban, 2011 [40]			x			Conference abstract (full study included in meta-analysis)
Wassenberg, 2004 [41]				x		Does not indicate fractures in ADHD patients
Yang, 2016 [42]		x		x		Excluded children previously diagnosed with ADHD; Assessed ADHD risk in those with fractures

### Characteristics of included studies

Two authors (H.S. and S.M.) assessed the quality of the five included studies and disagreements were discussed until consensus was reached. The characteristics of the included studies are given in Table 2. Three of the studies [9, 43, 44] contained a control group of children and adolescents who were not diagnosed with ADHD. Four of the studies [9, 43–45] indicated the percentage of ADHD patients who were male (between 76.5 and 81.6 %). Three of the studies [43, 45, 46] provided data on the anatomic location of fractures experienced by ADHD patients.

**Table. 2:** Characteristics of included studies

Study, Year	Country	Study Setting	Study Design	Duration	Sample Size		Age Range	Sex (of ADHD cohort)	Identification Method		Outcomes Reported	Fracture Types Reported
					**ADHD Group**	**Control Group**		**Male**	**ADHD**	**Fracture**		
Brehaut, 2003[9]	Canada	British Columbia (BC) Linked Health Dataset (BCLHD) and the BC Triplicate Prescription Program	Cohort (retro-spective)	7 years	16,806	1,010,067	0–19 years	81.6 %	Methylphenidate prescription	ICD-9-CM	Fractures; Open wounds; Poisoning/toxic effect; Intracranial; Concussion; Burns	Overall
Chou, 2014[43]	Taiwan	Longitudinal Health Insurance Database (LHID)	Cohort (retro-spective)	11 years	3,640	14,560	0–18 years	79.0 %	ICD-9-CM		Fractures	Skull, neck, ribs, and spine; Upper limb; Lower limb
Jacob, 2017[45]	Germany	Disease Analyzer database (IMS Health)	Nested case-control	6 years	27,880		6–17 years	76.5 %	ICD-10	ICD-10	Fractures	Forearm; Wrist and hand; Shoulder and upper arm; Foot and toe (except ankle); Lower leg (including ankle); Skull and face; Other
Raman, 2013[46]	United Kingdom	The Health Improvement Network (THIN)	Self-controlled case series	15.5 years	4,234		1–18 years		Read clinical classification system identification AND methylphenidate or dexamphetamine prescription	Read clinical classification system identification	Fractures; Intracranial; Traumatic complications; Sprains and strains; Superficial injury; Contusion; Open wound; Poisoning; Crushing injury; Foreign body in orifice; Burns; Other	Upper limb; Lower limb; Skull
van den Ban, 2013[44]	Nether-lands	PHARMO record linkage system (RLS)	Cohort (retro-spective)	11 years	1,289	7,332	0–18 years	79.8 %	Methylphenidate and atomextine prescription		Injuries or poisoning; Fractures; Intracranial; Open wounds	Overall

#### Outcomes

**Fig. 2:**
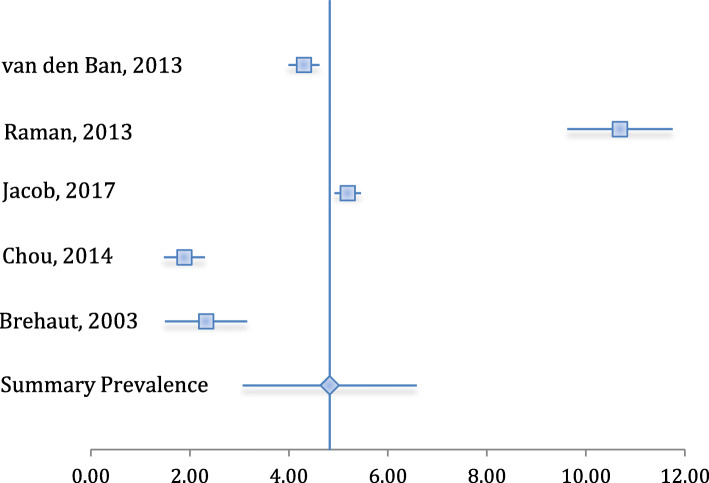
Forest plot of included studies

The summary es of the meta-analysis revealed a fracture prevalence of 4.83 %, with a 95 % CI between 3.07 and 6.58 (Table 3). The I^2^ score revealed a heterogeneity level of 66.63 %, a level that may be referred to as moderate-to-high [16]. The forest plot displaying the included studies and summary prevalence is given in Fig. 2.

**Table. 3:** Fracture prevalence outcomes, with 95 % CI

Study	Fractures (among ADHD)	Sample Size (ADHD)	Prevalence	CI lower	CI upper
Brehaut, 2003	723	16,806	4.30 %	3.99 %	4.62 %
Chou, 2014	389	3,640	10.69 %	9.62 %	11.75 %
Jacob, 2017	1,447	27,880	5.19 %	4.92 %	5.46 %
Raman, 2013	80	4,234	1.89 %	1.48 %	2.30 %
van den Ban, 2013	30	1,289	2.33 %	1.49 %	3.16 %
**Summary**			**4.83 %**	**3.07 %**	**6.58 %**

Two other outcomes were calculated using the available data from the included studies: fracture locations (Table 4) and fold-increase in fracture prevalence among the ADHD population (Table 5). It was determined, from the three studies that provided information on the locations of fractures, that the distribution of fractures among children and adolescents with ADHD is 69.62 % in the upper limbs, 22.85 % in the lower limbs, and 7.53 % in other skeletal regions (Table 4). Three of the studies in the meta-analysis included a control group design; therefore, by combining data solely from these studies, it was determined that there exists a 2.55-fold increase in the prevalence of fractures among children and adolescents with ADHD compared to those without ADHD (Table 5).

**Table. 4:** Fracture locations among children and adolescents with ADHD

Study	Overall	Upper Limb		Lower Limb		Other	
		Number	Percent	Number	Percent	Number	Percent
Brehaut, 2003	723^a^						
Chou, 2014	389	257	66.07 %	95	24.42 %	37	9.51 %
Jacob, 2017	1,447	1,026	70.90 %	320	22.10 %	101	7.00 %
Raman, 2013	80	51	63.75 %	23	28.75 %	6	7.50 %
van den Ban, 2013	30^a^						
**Summary**	**1,916**	**1,334**	**69.62 %**	**438**	**22.85 %**	**144**	**7.53 %**

^a^data not included in the summary numbers

**Table. 5:** Prevalence of fractures in ADHD group versus control group

Study	ADHD Group			Control Group			Fold Increase
	**Sample Size**	**Fractures**	**Prevalence**	**Sample Size**	**Fractures**	**Prevalence**	
Brehaut, 2003	16,806	723	4.30 %	1,010,067	20,025	1.98 %	**2.17**
Chou, 2014	3,640	389	10.69 %	14,560	1,188	8.16 %	**1.31**
Jacob, 2017	27,880^a^	1,447^a^	5.19 %^a^				
Raman, 2013	4,234^a^	80^a^	1.89 %^a^				
van den Ban, 2013	1,289	30	2.33 %	7,332	90	1.23 %	**1.90**
**Summary**	**21,735**	**1,142**	**5.25 %**	**1,031,959**	**21,303**	**2.06 %**	**2.55**

^a^data not included in the summary numbers

## Discussion

ADHD is a significant neurodevelopment disorder with a prevalence of 5 % among children and adolescents [4]. Bone fractures account for 25 % of accidents and injuries among children and adolescents [5]. Considering the hallmark characteristics of inattention, hyperactivity, and impulsivity in ADHD [1], it is critical to examine the prevalence of bone fractures in this population.

The meta-analysis revealed that the prevalence of fractures among children and adolescents diagnosed with ADHD is 4.83 % (95 % CI: 3.07 to 6.58). A subset of the studies included in the meta-analysis also determined a 2.55-fold increase in the prevalence of fractures among this group when compared to a control group. Another subset of studies discovered the distribution of fractures among the population with ADHD to be in the upper limb (69.62 %), lower limb (22.85 %), and other anatomic regions (7.53 %).

There is an array of possible reasons for injuries and fractures in children with ADHD that may be attributed to the characteristic symptoms of the disorder. For example, parents report that impulsive children do not think before they act, can show poor judgment, and seek immediate gratification [47]. Another study found that while children with ADHD were able to identify hazards, they anticipate less severe consequences to their behaviors [48]. Pastor and Reuben [30] determined that although children with ADHD experience more injuries, their modes of injury are similar to children without ADHD. Therefore, characteristics of ADHD may make these children more prone to experiencing injuries and fractures even when they are in similar settings and situations to their counterparts without ADHD.

## Limitations

The authors have identified several limitations to this meta-analysis including its heterogeneity and the causes of this heterogeneity. Heterogeneity may be clinical, methodological, or statistical. Clinical heterogeneity may stem from differences in the characteristics of the populations in the study [14]. As was previously discussed, different diagnostic guidelines will identify varying subsets of children and adolescents as having ADHD. Therefore, since different methods of diagnosis were used among the studies, those identified with ADHD may have variable severities of the disorder, which may impact the prevalence of fractures.

Another source of clinical heterogeneity stems from whether medications were used to treat ADHD. In those studies where children and adolescents were identified through their diagnosis of ADHD and not the prescription of medications, it was not known if those experiencing fractures were using pharmacologic interventions for ADHD. It has been reported that the use of both stimulant and non-stimulant medications to treat ADHD is associated with a decrease in the number of bone fractures among patients with ADHD [31]. Another study revealed a 25 % decrease in the risk of fractures in those who took methylphenidate for 180 days or longer [20]. Therefore, the prevalence of fractures between the studies may have been affected by the rate of pharmacological interventions.

Methodological heterogeneity can arise from variations in study qualities or differences in study designs [14]. All of the studies included in this meta-analysis were found to be similar in quality, as discussed in the quality assessment section. Additionally, all study designs were composed of a retrospective method that used a national or regional database. However, the studies differed in their time periods, which may have led to methodological heterogeneity. Both the clinical and methodological heterogeneities may have given rise to the statistical heterogeneity observed in the meta-analysis.

Another shortcoming of the meta-analysis is having only aggregate numbers of fractures among patients with ADHD. It is not known if a patient experienced multiple fractures in one injury event, if a patient had a series of fractures over time, or if each patient had one fracture each to compose the overall fracture numbers. It was also not possible to stratify the prevalence findings by age subgroups or by gender.

## Future implications

If studies become available, future meta-analyses should be completed on prospective study designs that can follow children and adolescents with ADHD over time to determine who experiences what types of fractures, from what sources of injury, and at what age. The participants’ usage of medication for the treatment of ADHD should also be tracked.

A thorough subgroup analysis is required in future research, especially by age and gender. The modes of injury and bone integrity for a young child may be very different than that of an adolescent. The ability to stratify bone fractures by age can inform measures aimed at preventing injuries that are most effectively aimed at specific age groups. Some studies that have assessed fractures by gender in children and adolescents with ADHD have determined a higher rate among girls than boys when compared to control groups of girls and boys without ADHD [49]. With the knowledge that such differences exist, future research needs to quantify this difference and qualify it in a way that can impact the lives of patients.

## Conclusions

Bone fractures in children and adolescents are an important concern for public health because they have considerable influence on the activities and daily living of affected children [50]. With an ADHD prevalence of 5 % among children and adolescents and a fracture prevalence of nearly 5 % among this population, understanding bone fractures among children and adolescents with ADHD is of importance in improving health and healthcare delivery. This understanding is most critical to parents who care for and attempt to decrease the occurrence of fractures in their children, practitioners who educate their patients in an attempt to prevent injury or who treat these children once they have experienced a fracture, and to policy makers whose obligation it is to create healthy and safe environments that promote the health of all children and adolescents.

## Data Availability

This article is a systematic review and meta-analysis; therefore, all data is available through published studies as described in the article.

## Abbreviations

ADHD: Attention-deficit/hyperactivity disorder
es: Effect size
DSM: Diagnostic and Statistical Manual of Mental Disorders
ICD: International Classification of Diseases
PRISMA: Preferred Reporting Items for Systematic Reviews and Meta-Analyses
JBI: Joanna Briggs Institute
CI: Confidence interval

## References

[1] Thapar A, Cooper M (2016). Attention deficit hyperactivity disorder. The Lancet.

[2] Polanczyk GV, Willcutt EG, Salum GA, Kieling C, Rohde LA (2014). ADHD prevalence estimates across three decades: an updated systematic review and meta-regression analysis. International journal of epidemiology..

[3] Skounti M, Philalithis A, Galanakis E (2007). Variations in prevalence of attention deficit hyperactivity disorder worldwide. Eur J Pediatrics.

[4] American Psychiatric Association (2013). Diagnostic and statistical manual of mental disorders.

[5] Cooper C, Dennison EM, Leufkens HG, Bishop N, van Staa TP (2004). Epidemiology of childhood fractures in Britain: a study using the general practice research database. J Bone Miner Res.

[6] Powell EC, Tanz RR (2002). Adjusting our view of injury risk: the burden of nonfatal injuries in infancy. Pediatrics..

[7] Wilkins KE, Aroojis AJ, Beaty JH, Kasser JR (2005). Incidence of Fractures in Children. Rockwood and Wilkins’ Fractures in Children.

[8] Barkley RA (2001). Accidents and attention-deficit/hyperactivity disorder. Econ Neurosci.

[9] Brehaut JC, Miller A, Raina P, McGrail KM (2003). Childhood behavior disorders and injuries among children and youth: a population-based study. Pediatrics..

[10] Moher D, Liberati A, Tetzlaff J, Altman DG. Prisma Group. Preferred reporting items for systematic reviews and meta-analyses: the PRISMA statement. PLoS medicine. 2009;6(7):e1000097. 10.1371/journal.pmed.1000097 PMC2707599 19621072

[11] Ruel JM, Hickey CP (1992). Are too many children being treated with methylphenidate?. The Canadian Journal of Psychiatry.

[12] Joanna Briggs Institute. (2017). The Joanna Briggs Institute Critical Appraisal tools for use in JBI Systematic Reviews: Checklist for Prevalence Studies. South Australia, Australia: Joanna Briggs Institute. Retrieved from http://joannabriggs.org.

[13] Neyeloff JL, Fuchs SC, Moreira LB (2012). Meta-analyses and Forest plots using a microsoft excel spreadsheet: step-by-step guide focusing on descriptive data analysis. BMC Res Notes.

[14] Joanna Briggs Institute. (2014). Joanna Briggs Institute Reviewers’ Manual: 2014 Edition. South Australia, Australia: Joanna Briggs Institute. Retrieved from http://joannabriggs.org.

[15] Higgins JP. Commentary. Heterogeneity in meta-analysis should be expected and appropriately quantified. International journal of epidemiology. 2008;37(5):1158-60. 10.1093/ije/dyn204 18832388

[16] Higgins JP, Thompson SG, Deeks JJ, Altman DG (2003). Measuring inconsistency in meta-analyses. Bmj..

[17] Higgins JP, Thompson SG, Spiegelhalter DJ (2009). A re-evaluation of random-effects meta-analysis. Journal of the Royal Statistical Society: Series A (Statistics in Society)..

[18] Ayaz M, Ayaz AB, Soylu N (2015). Socio-Demographic and behavioral factors related to unintentional injuries in preschool children diagnosed to have attention-deficit/hyperactivity disorder. Klinik Psikofarmakoloji Bülteni-Bulletin of Clinical Psychopharmacology..

[19] Cairney J (2014). Deficits in attention, motor control, and perception and increased risk of injury in children. Developmental Medicine Child Neurology.

[20] Chen VC, Yang YH, Liao YT, Kuo TY, Liang HY, Huang KY, Huang YC, Lee Y, McIntyre RS, Lin TC (2017). The association between methylphenidate treatment and the risk for fracture among young ADHD patients: A nationwide population-based study in Taiwan. PloS one..

[21] Duramaz A, Yilmaz S, Ziroğlu N, Duramaz BB, Bayram B, Kara T (2019). The role of psychiatric status on pediatric extremity fractures: a prospective analysis. Eur J Trauma Emerg Surg.

[22] Erdogan M, Desteli EE, Imren Y, Yuce M, Buyukceran I, Karadeniz E (2014). Is attention deficit and hyperactivity disorder a risk factor for sustaining fractures of proximal humerus. Acta Chir Orthop Traumatol Cech..

[23] Guo NW, Lin CL, Lin CW, Huang MT, Chang WL, Lu TH, Lin CJ (2016). Fracture risk and correlating factors of a pediatric population with attention deficit hyperactivity disorder: a nationwide matched study. Journal of Pediatric Orthopaedics B..

[24] Guy JA, Knight LM, Wang Y, Jerrell JM (2016). Factors associated with musculoskeletal injuries in children and adolescents with attention-deficit/hyperactivity disorder. The primary care companion for CNS disorders..

[25] Hellgren L, Gillberg C, Gillberg IC, Enerskog I (1993). Children with deficits in attention, motor control and perception (DAMP) almost grown up: general health at 16 years. Developmental Medicine Child Neurology.

[26] Hurtig T, Ebeling H, Jokelainen J, Koivumaa-Honkanen H, Taanila A (2016). The association between hospital-treated injuries and ADHD symptoms in childhood and adolescence: a follow-up study in the northern Finland birth cohort 1986. Journal of attention disorders.

[27] Lu YY, Wang MY, Wei IH, Lin CC, Huang CC. Tourette syndrome increases risk of bone fractures: a population-based cohort study. European child & adolescent psychiatry. 2017 May 1;26(5):531-9. 10.1007/s00787-016-0916-4 27807804

[28] Merrill RM, Lyon JL, Baker RK, Gren LH (2009). Attention deficit hyperactivity disorder and increased risk of injury. Advances in medical sciences.

[29] Ozer K, Gillani S, Williams A, Hak DJ (2010). Psychiatric risk factors in pediatric hand fractures. Journal of Pediatric Orthopaedics..

[30] Pastor PN, Reuben CA (2006). Identified attention-deficit/hyperactivity disorder and medically attended, nonfatal injuries: US school-age children, 1997–2002. Ambul Pediatr.

[31] Perry BA, Archer KR, Song Y, Ma Y, Green JK, Elefteriou F, Dahir KM (2016). Medication therapy for attention deficit/hyperactivity disorder is associated with lower risk of fracture: a retrospective cohort study. Osteoporosis International.

[32] Prasad V, West J, Sayal K, Kendrick D. Injury among children and young people with and without attention-deficit hyperactivity disorder in the community: The risk of fractures, thermal injuries, and poisonings. Child: Care,Health Dev. 2018;44(6):871-8. 10.1111/cch.12591 30039608

[33] Rowe R, Maughan B, Goodman R (2004). Childhood psychiatric disorder and unintentional injury: findings from a national cohort study. J Pediatr Psychol.

[34] Schermann H, Ankory R, Shlaifer A, Dolkart O, Rotman D, Yoffe V, Karakis I, Chechik O (2019). Lower risk of stress fractures in young adults with ADHD under chronic treatment with methylphenidate. Bone.

[35] Schermann H, Gurel R, Ankory R, Kadar A, Yoffe V, Snir N, Sternheim A, Karakis I (2018). Lower risk of fractures under methylphenidate treatment for ADHD: A dose–response effect. J Orthop Res.

[36] Siwani R, Tombers NM, Rieck KL, Cofer SA (2014). Comparative analysis of fracture characteristics of the developing mandible: the Mayo Clinic experience. International journal of pediatric otorhinolaryngology..

[37] Tai YM, Gau SS, Gau CS (2013). Injury-proneness of youth with attention-deficit hyperactivity disorder: a national clinical data analysis in Taiwan. Research in developmental disabilities..

[38] Uslu MM, Uslu R (2008). Extremity fracture characteristics in children with impulsive/hyperactive behavior. Arch Orthopaedic Trauma Surg.

[39] Uslu M, Uslu R, Eksioglu F, Ozen NE (2007). Children with fractures show higher levels of impulsive-hyperactive behavior. Clin Orthop Relat Res.

[40] Van den Ban E, Souverein P, Meijer W, Van Engeland H, Swaab H, Egberts T, Heerdink E. 307. Accident Proneness among Youth Treated with ADHD Medication. Pharmacoepidemiology and Drug Safety 2011;20:133–4.

[41] Wassenberg R, Max JE, Koele SL, Firme K (2004). Classifying psychiatric disorders after traumatic brain injury and orthopaedic injury in children: adequacy of K-SADS versus CBCL. Brain Injury..

[42] Yang LY, Huang CC, Chiu WT, Huang LT, Lo WC, Wang JY (2016). Association of traumatic brain injury in childhood and attention-deficit/hyperactivity disorder: a population-based study. Pediatric research.

[43] Chou IC, Lin CC, Sung FC, Kao CH (2014). Attention-deficit‐hyperactivity disorder increases risk of bone fracture: a population‐based cohort study. Developmental Medicine Child Neurology.

[44] van den Ban E, Souverein P, Meijer W, Van Engeland H, Swaab H, Egberts T, Heerdink E (2014). Association between ADHD drug use and injuries among children and adolescents. European child & adolescent Psychiatry..

[45] Jacob L, Kostev K (2017). Impact of attention deficit hyperactivity disorder therapy on fracture risk in children treated in German pediatric practices. Osteoporosis International..

[46] Raman SR, Marshall SW, Haynes K, Gaynes BN, Naftel AJ, Stürmer T. Stimulant treatment and injury among children with attention deficit hyperactivity disorder: an application of the self-controlled case series study design. Injury Prev 2013;19(3):164 – 70. 10.1136/injuryprev-2012-040483 23143347

[47] Loder RT, Warschausky S, Schwartz EM, Hensinger RN, Greenfield ML (1995). The psychosocial characteristics of children with fractures. J Pediatr Orthop.

[48] Farmer JE, Peterson L (1995). Injury risk factors in children with attention deficit hyperactivity disorder. Health psychology.

[49] Guo NW, Lin CL, Lin CW, Huang MT, Chang WL, Lu TH, Lin CJ. Fracture risk and correlating factors of a pediatric population with attention deficit hyperactivity disorder: a nationwide matched study. Journal of Pediatric Orthopaedics B 2016;25(4):369 – 74. 10.1097/BPB.0000000000000243 26523534

[50] Kopjar B, Wickizer TM (1998). Fractures among children: incidence and impact on daily activities. Injury Prevention.

